# A retrospective case-control study of Da Vinci robotic versus laparoscopic radical gastrectomy for gastric cancer under an enhanced recovery after surgery protocol

**DOI:** 10.3389/fonc.2026.1793088

**Published:** 2026-05-20

**Authors:** Wen-hao Sun, Hao-di Wang, Gang Wang, Ming-yue Shao, Zheng-ming Deng, Zhi-wei Jiang

**Affiliations:** 1 Affiliated Hospital of Nanjing University of Chinese Medicine, Nanjing, China; 2 Nanjing University of Chinese Medicine, Nanjing, China

**Keywords:** Da Vinci robotic system, Enhanced Recovery After Surgery, gastric cancer, laparoscopy, minimally invasive surgery, multivariate analysis, prolonged hospitalisation, radical gastrectomy

## Abstract

**Objective:**

To compare the safety and short-term efficacy of radical gastrectomy using the Da Vinci fully robotic system versus laparoscopic surgery under a standardised enhanced recovery after surgery (ERAS) protocol, with multivariate adjustment for baseline differences in tumour stage and nutritional status.

**Methods:**

A retrospective case-control analysis of 259 patients (RTG: n=121; LTG: n=138) undergoing gastric cancer radical surgery at Jiangsu Provincial Hospital of Traditional Chinese Medicine (August 2018–July 2022) under a uniform institutional ERAS protocol. Univariate comparisons were supplemented by multivariate regression analyses adjusting for pathological TNM stage, preoperative albumin, age, and BMI.

**Results:**

The RTG group had significantly earlier pathological TNM staging (Stage I: 40.5% vs. 21.7%, p=0.003) and higher preoperative albumin (40.8 vs. 39.05 g/L, p<0.001). In univariate analysis, the RTG group demonstrated shorter median postoperative hospital stay (6 vs. 9 days, p<0.001), a markedly lower rate of prolonged hospitalisation >14 days (1.7% vs. 14.5%, OR = 10.1, p=0.001), earlier first oral intake (6 vs. 42.75 h, p<0.001), earlier first ambulation (20 vs. 38 h, p<0.001), fewer ICU admissions (5.0% vs. 17.4%, p=0.002), a higher rate of adequate lymph node dissection (≥10 groups: 72.7% vs. 44.2%, p<0.001), a lower rate of clinically significant blood loss (>100 ml: 2.5% vs. 19.6%, p<0.001), and significantly better preservation of postoperative albumin on day 1 (Δ −2.0 vs. Δ −3.9 g/L, p=0.001) and day 5 (Δ −0.7 vs. Δ −3.2 g/L, p<0.001). The largest recovery gap was observed in total gastrectomy (7 vs. 11 days, p=0.002) despite equal operative time (285 vs. 288 min, p=0.969). In multivariate regression adjusting for all covariates, the robotic approach remained independently associated with all primary recovery outcomes (hospital stay β=−3.25 days, first liquid intake β=−41.71 h, first ambulation β=−16.45 h, drain duration β=−1.79 days; all p<0.001). The overall complication rate did not differ significantly (5.0% vs. 7.2%, OR = 0.68, 95% CI 0.24–1.94, p=0.606); *post-hoc* power was 11.6%, indicating the comparison was statistically inconclusive.

**Conclusion:**

Robotic radical gastrectomy within a uniform ERAS protocol is independently associated with superior short-term postoperative recovery — including shorter hospitalisation, earlier oral intake, better nutritional preservation, and higher-quality lymph node dissection — compared to laparoscopic surgery, an advantage that persists after multivariate adjustment for tumour stage and is consistent across all subgroups examined. Multi-centre prospective trials are required to confirm long-term oncological outcomes.

## 1 Introduction

Gastric cancer remains a major global health burden, ranking fifth in incidence and fifth in cancer-related mortality worldwide in 2022, with approximately 968,000 new cases and 660,000 deaths annually (1). East Asia—and China in particular—bears a disproportionate share of this burden, accounting for nearly half of global cases and deaths (2), and modelling studies project that the absolute number of gastric cancer cases will continue to rise through 2040 due to population aging despite declining age-standardised incidence rates (3). Current international guidelines uniformly recommend radical gastrectomy with D2 lymphadenectomy as the cornerstone of curative treatment for locally advanced disease (4, 5), most commonly delivered within an Enhanced Recovery After Surgery (ERAS) pathway designed to attenuate perioperative stress and accelerate functional recovery (6). Since the first laparoscopy-assisted gastrectomy reported by Kitano et al. in 1994 (7), minimally invasive surgery has transformed the operative management of gastric cancer (8). Long-term evidence from three large randomised trials—CLASS-01 in China (9), JLSSG0901 in Japan (10), and KLASS-02 in Korea—has now established the oncological non-inferiority of laparoscopic distal gastrectomy compared with open surgery for both early and locally advanced disease. Yet conventional laparoscopy still faces intrinsic technical constraints in the execution of a radical D2 dissection: a two-dimensional operative field, limited instrument degrees of freedom, physiological hand tremor, and—most critically—the need for an auxiliary abdominal incision for extracorporeal gastrointestinal reconstruction.The Da Vinci robotic surgical system was designed to address precisely these limitations. Three-dimensional high-definition magnified visualisation, seven-degree-of-freedom wristed instruments with tremor filtration, and a stable operating platform together enable complex intracorporeal suturing and anastomosis (11, 12). These capabilities are particularly relevant for dissection of lymph node stations 9, 10, 11, and 14—the suprapancreatic and splenic hilar regions that are technically demanding under laparoscopy—and for fully intracorporeal digestive tract reconstruction, which eliminates the auxiliary laparotomy incision otherwise required in conventional laparoscopic gastrectomy. Among all technical modifications introduced by the robotic platform, this elimination of the auxiliary incision is arguably the single change most likely to reduce surgical trauma and accelerate recovery (13). Randomised evidence comparing robotic and laparoscopic gastrectomy has accumulated rapidly in the past five years. The two-centre Japanese trial by Ojima et al. found similar rates of intra-abdominal infectious complications but significantly lower drain amylase levels with the robotic approach (14). The Chinese single-centre trial by Lu et al. demonstrated shorter postoperative recovery and comparable long-term oncological outcomes in favour of robotic distal gastrectomy (15), with 3-year disease-free survival results recently reported (16). A Brazilian trial by Ribeiro et al. confirmed the feasibility of robotic gastrectomy compared with open surgery (17). These randomised findings are consistent with Guerrini et al.'s large meta-analysis of 17,000+ patients (18), a recent Bayesian network meta-analysis that placed robotic gastrectomy at the top of the ranking for short-term recovery outcomes (19), and the largest Chinese multicentre cohort study of 5,402 patients reporting modest but statistically significant advantages for robotic surgery across multiple perioperative endpoints (20). A critical methodological limitation of most existing comparative studies, however, is inadequate control for the baseline imbalance in pathological tumour stage that commonly exists between robotic and laparoscopic groups in observational data. Because robotic gastrectomy is typically adopted first for earlier-stage, lower-risk cases, robotic cohorts often contain a higher proportion of Stage I patients—who recover faster regardless of surgical technique. Without appropriate statistical adjustment, this selection bias can substantially overestimate the benefits of the robotic approach. Furthermore, the relationship between surgical approach and uniform ERAS compliance has rarely been addressed as a distinct question: most prior comparisons either lack a standardised ERAS protocol or do not quantify the independent contribution of the surgical platform after adjustment for staging and nutritional baseline.The present study directly addresses these two limitations. We retrospectively compare robotic and laparoscopic radical gastrectomy performed by a single experienced surgical team within a uniform institutional ERAS protocol, incorporating multivariate regression for confounding adjustment, multiple pre-specified subgroup analyses, and novel analyses of postoperative albumin trajectory, prolonged hospitalisation, and clinically significant intraoperative blood loss thresholds.

## 2 Patients and methods

### 2.1 Study design and patient selection

This is a retrospective case-control study evaluating the clinical safety and short-term efficacy of robotic-assisted versus laparoscopic radical gastrectomy for gastric cancer. Medical records of all patients undergoing gastric cancer radical surgery at Jiangsu Provincial Hospital of Traditional Chinese Medicine between August 2018 and July 2022 were retrospectively reviewed. A total of 527 consecutive cases were initially screened.

Patients were excluded if they met any of the following criteria: (1) ASA physical status Grade IV (n=8); (2) intraoperative stoma creation (n=13); (3) postoperative pathological stage ≥ IV (n=9); (4) combined resection of other organs due to tumour invasion of adjacent structures, or abdominal closure (n=35); (5) prior endoscopic resection with no residual tumour, pathological complete response after preoperative chemotherapy, or pathological insufficiency (n=29); (6) open surgery (n=85); (7) inability to comply with the postoperative ERAS protocol (n=22); (8) malignant tumour of the gastric remnant (n=8); (9) postoperative pathological diagnosis of non-adenocarcinoma histology (n=59). The exclusion of patients unable to comply with the ERAS protocol (n=22) was applied equally to both groups and ensured that all analysed patients received the full standardised perioperative pathway, thereby reducing the risk of protocol non-compliance as a confounding source.

**Figure 1:**
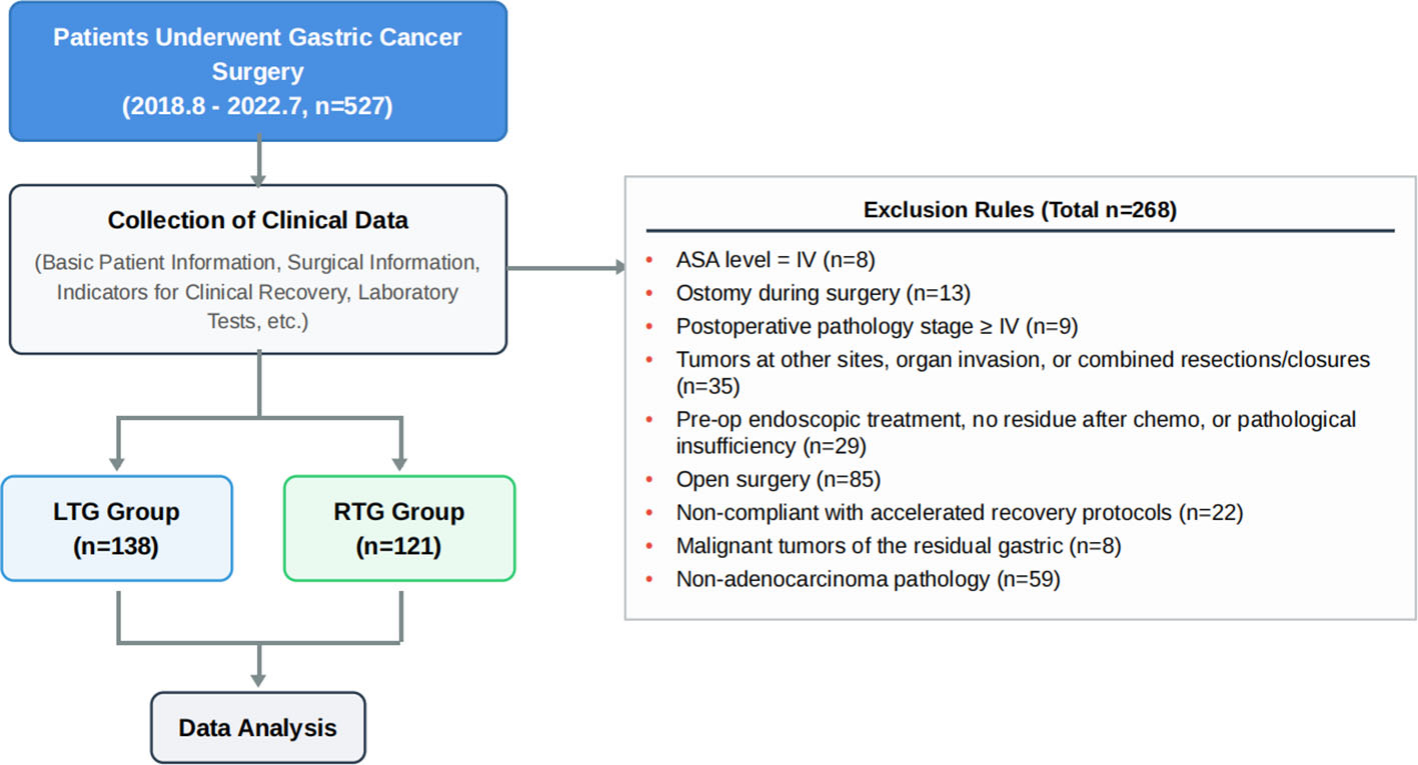
Flowchart of patient selection.

After all exclusions, 121 patients were enrolled in the robotic group (RTG) and 138 in the laparoscopic group (LTG). Patients were allocated to each group based on their own operative preference: after a structured preoperative consultation in which both surgical approaches were explained, each patient voluntarily chose their preferred technique and signed a procedure-specific informed consent form. The study was conducted in accordance with the Declaration of Helsinki and approved by the Ethics Committee of Jiangsu Provincial Hospital of Traditional Chinese Medicine (approval number: 2021NL-149-01). The overall patient selection process is illustrated in **Figure 1**.

### 2.2 Surgical procedures and quality control

**Figure 2:**
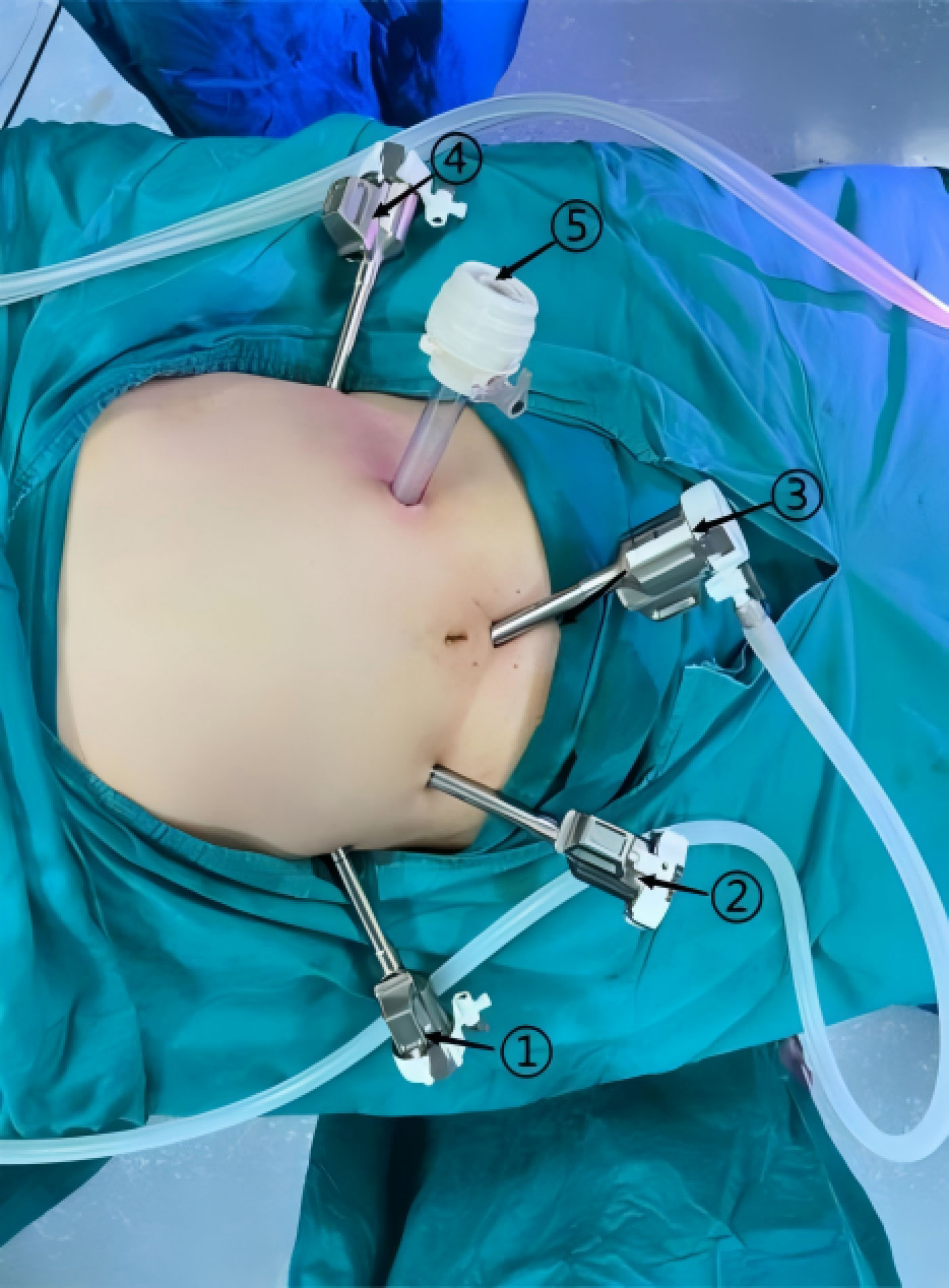
Schematic diagram of robotic arms and Trocars positioning.

**Figure 3:**
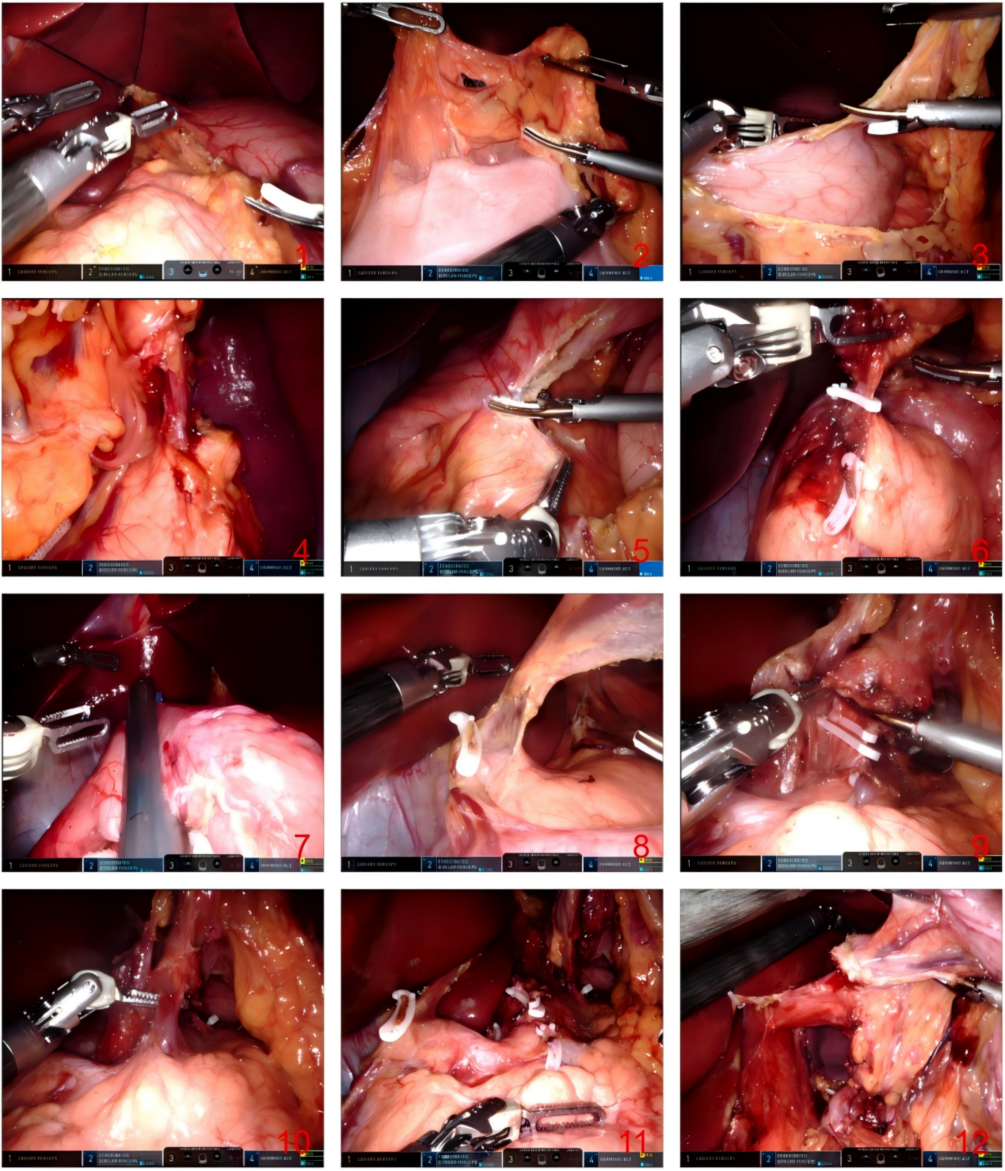
Operation procedure. [(1) Cut the hepatogastric ligament and suspend the left lobe of the liver; 2) Cut the appetising colic ligament; (3) The gastrocolic ligaments were severed along the greater curvature of the stomach; (4) Left gastroepiploic lymph node was dissected; (5) Free the duodenum along the greater curvature of the stomach; (6) Lymph nodes around the right arteriovenous gastric omentum were dissected. (7) Duodenal dissection; (8) Lymph nodes around the right gastric artery were dissected; (9) Severed left gastric artery; (10) Severed left gastric vein; (11) Lymph nodes in groups 7/8/9 were cleared; (12) Free oesophagus].

All procedures were performed by the surgical team led by Professor Zhi-wei Jiang. Prior to commencement of this study in August 2018, the team had independently completed over 200 robotic gastrointestinal procedures. The specific surgical procedures and techniques used by our team are detailed below. For the robotic group, following general anesthesia with endotracheal intubation, the patient was placed in a supine position with head-up and left-side-up tilt. The surgical field was sterilized with an iodine-based solution and draped with sterile towels. Trocar placement: A 1 cm infraumbilical incision was made to introduce a 12 mm trocar, which served as the camera port (port ③, corresponding to the third robotic arm). Carbon dioxide (CO₂) pneumoperitoneum was established at a pressure of 12–14 mmHg. Additional trocars were placed as follows: at the midclavicular line, 1–2 cm below the xiphoid process bilaterally, 8 mm ports were inserted (port ①, corresponding to the first robotic arm, and port ④, corresponding to the fourth robotic arm). On the left and right sides at the midclavicular line, 12 mm and 8 mm ports were placed, respectively (port ②, corresponding to the second robotic arm). A supplementary 12 mm assistant port (port ⑤) was also used. Details are illustrated in **Figure 2**. In accordance with the Japanese Gastric Cancer Treatment Guidelines and based on tumor location, size, and stage, either total gastrectomy, proximal gastrectomy, or distal gastrectomy with D2 lymph node dissection was performed using standard laparoscopic techniques. Digestive tract reconstruction was completed under robotic assistance. Following distal gastrectomy, either a Billroth I anastomosis (remnant stomach to duodenum) or a Billroth II anastomosis (remnant stomach to jejunum) was constructed. After proximal gastrectomy, a gastrojejunostomy was performed. For total gastrectomy, both a gastrojejunostomy and a jejunojejunostomy were carried out. The resected specimen was placed in a retrieval bag and positioned below the umbilicus. The robotic camera port incision was extended, the specimen bag was extracted, and the incision was subsequently closed (the standardized robotic surgical workflow is illustrated in **Figure 3**). The surgical approach in the laparoscopic group was analogous to that of the robotic group, with the following principal differences: (1) Trocar distribution was slightly modified. The positions of the two right-sided abdominal trocars were typically exchanged: the 12 mm assistant port was placed at the right anterior axillary line, 1–2 cm inferior to the costal margin. Given the greater dexterity and precision of the right hand in most individuals, the larger assistant port was routinely positioned on the right side. (2) During digestive tract reconstruction in the laparoscopic procedure, an additional 9 cm midline upper abdominal incision was created to gain access to the peritoneal cavity, and reconstruction was performed extracorporeally using a tubular stapler and a cutting-closing device. No intraoperative conversions from robotic to laparoscopic or open surgery occurred throughout the study period. Operative time stability across the study period further confirms that the learning curve had been overcome prior to enrolment. The robotic group (RTG) used a five-trocar configuration with fully intracorporeal digestive tract reconstruction, eliminating the need for any auxiliary abdominal incision. The laparoscopic group (LTG) used an analogous trocar configuration but required an additional 9 cm upper midline incision for extracorporeal reconstruction.

### 2.3 ERAS protocol

Both groups were managed under the same institutional ERAS protocol, implemented since 2016. This protocol standardised: (1) preoperative patient education; (2) nutritional preparation (no mechanical bowel preparation; carbohydrate-rich drink 2 hours preoperatively); (3) goal-directed intraoperative fluid therapy and normothermia; (4) multimodal postoperative analgesia (sufentanil-based PCIA, scheduled NSAIDs, paracetamol; target VAS ≤3); (5) early urinary catheter removal (targeting within 24 hours); (6) early supervised ambulation (day 1 postoperatively); (7) early oral feeding reinstatement (clear liquids on resumption of bowel sounds); (8) drain removal when output <50 ml/day of serous fluid; and (9) routine thromboprophylaxis. The significant difference in gastric tube retention (RTG 13.2% vs. LTG 45.7%, p<0.001) reflects the reduced need for gastrointestinal decompression after intracorporeal reconstruction, not a protocol difference. This protocol is consistent with the ERAS Society 2014 gastrectomy guideline (6) and aligns with the perioperative management standards used in major comparative trials such as LOGICA (21).

### 2.4 Outcome measures

Primary outcomes: postoperative hospital stay, time to first oral liquid intake, time to first ambulation, and overall postoperative complication rate. Secondary outcomes included: intraoperative blood loss (as continuous variable and binary threshold >100 ml), operative time, lymph node yield (as continuous variable and binary threshold >=10 groups), time to first flatus, urinary catheter and drain duration, VAS scores, ICU rate, Clavien-Dindo classification, perioperative laboratory values, postoperative albumin trajectory (delta albumin from baseline), prolonged hospitalisation rate (>14 days), and drain removal within 5 days.

### 2.5 Statistical analysis

Statistical analysis was performed using SPSS Statistics version 26.0 (IBM Corporation). Shapiro-Wilk tests confirmed that all primary continuous outcome variables were non-normally distributed (all p<0.001); accordingly, these are presented as median (IQR) and compared by Wilcoxon rank-sum test. Variables with approximate normality are presented as mean ± SD and compared by independent-samples t-test. Categorical variables are compared by chi-square test or Fisher exact test. Laboratory indices were compared at each time point independently using the appropriate between-group test (t-test or Wilcoxon rank-sum test), not by repeated-measures ANOVA. All tests were two-sided; p<0.05 was considered significant.

To control for identified baseline imbalances in pathological TNM stage and preoperative albumin, multivariate analyses were performed. Multivariate linear regression (β with 95% CI) was applied to continuous recovery outcomes (hospital stay, time to first liquid intake, first ambulation, drain duration). Multivariate binary logistic regression (OR with 95% CI) was applied to overall complication occurrence. All models included: pathological TNM stage (categorical, Stage I as reference), preoperative albumin (continuous), age (continuous), and BMI (continuous). For the complication comparison, OR with 95% CI is reported, and *post-hoc* power was calculated to characterise the reliability of the negative finding. Spearman rank correlation was used to assess associations between recovery variables. All subgroup analyses were pre-specified by TNM stage, surgical procedure type, BMI category, and ASA grade.

## 3 Results

### 3.1 Patient characteristics

**Figure 4:**
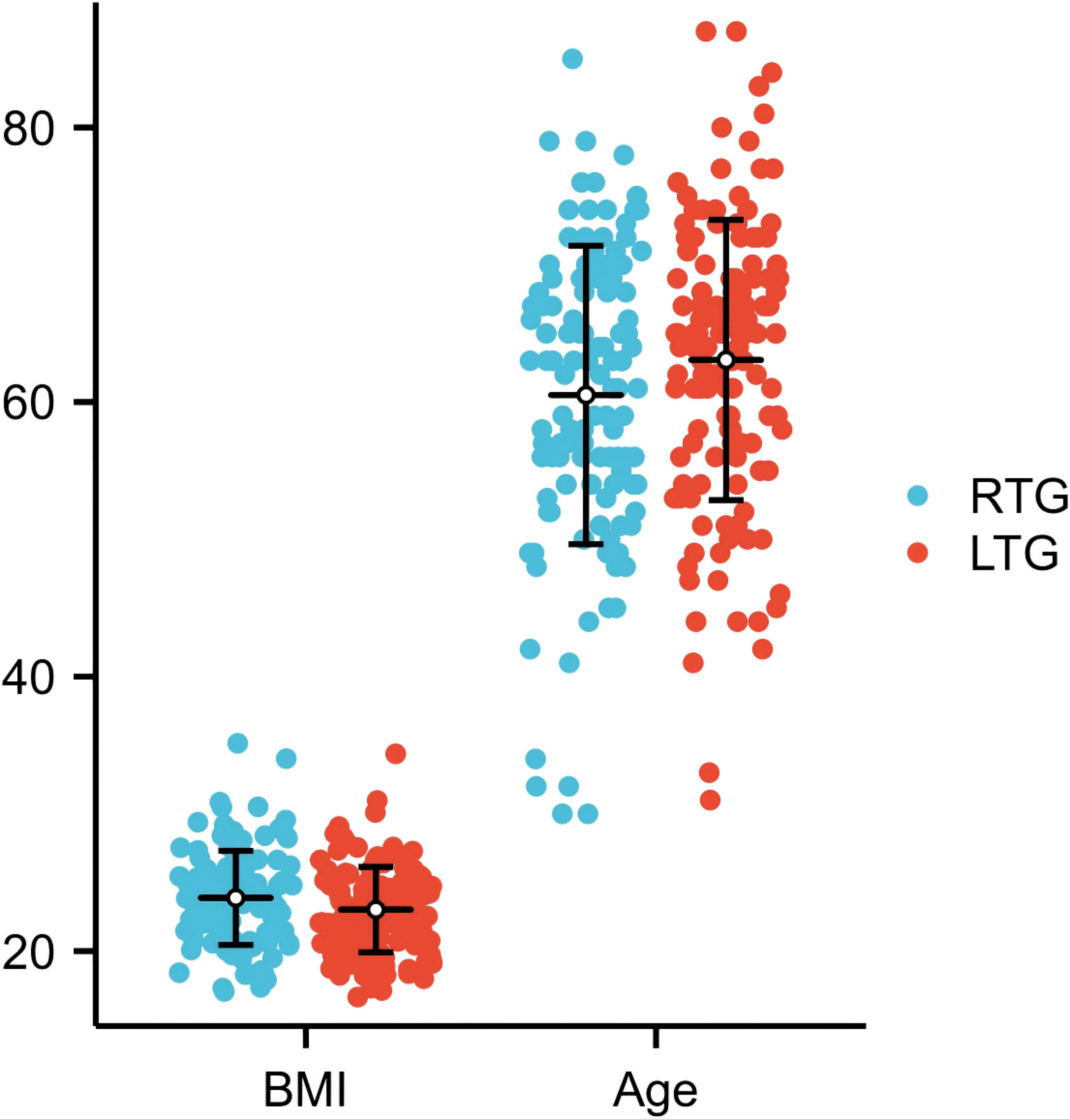
BMI and age information of all patients.

**Figure 5:**
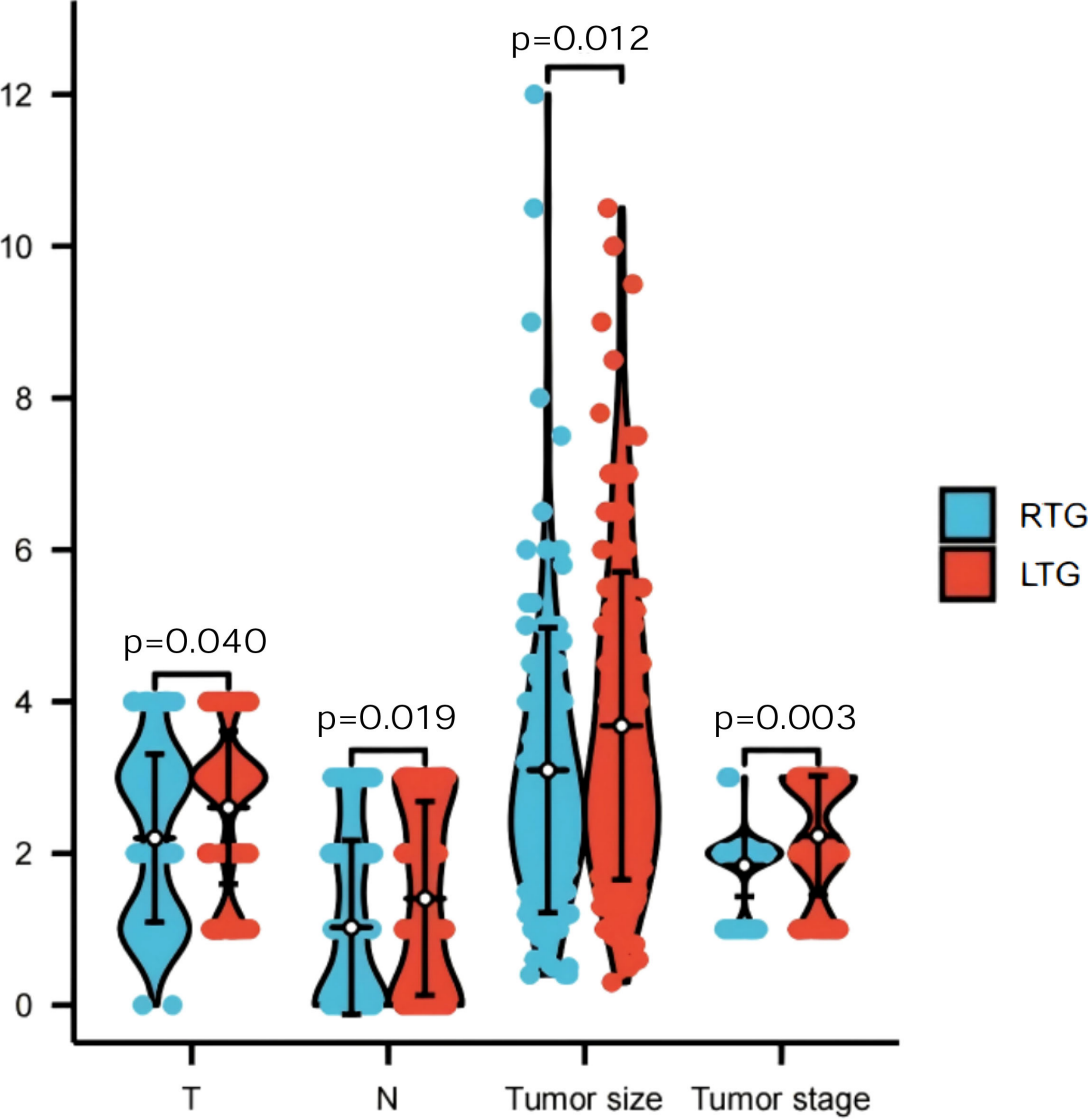
Tumour staging status.

A total of 259 patients were enrolled. Baseline characteristics were broadly balanced between groups (**Table 1**), with no statistically significant differences in sex, age, BMI, comorbidity burden, preoperative chemotherapy, or ASA score. Preoperative hypoalbuminaemia (albumin <35 g/L) was present in 7.4% of RTG patients versus 12.3% in the LTG group (p=0.172), confirming no significant difference in baseline nutritional risk by this criterion. Data distributions are shown in **Figures 4**, **5**.

**Table 1:** General patient characteristics (RTG vs. LTG).

Variables	RTG (n=121)	LTG (n=138)	Statistic	P value
Age, years (median, IQR)	62 (54, 69)	65 (57, 69)	Z = −1.723	0.085
Sex, n (%)			χ² = 1.291	0.271
Male	91 (75.2)	95 (68.8)		
Female	30 (24.8)	43 (31.2)		
BMI, kg/m² (median, IQR)	23.88 (21.26, 25.48)	23.13 (20.71, 24.98)	Z = −1.924	0.054
Underlying comorbidity, n (%)			χ² = 0.037	0.898
No	74 (61.2)	86 (62.3)		
Yes	47 (38.8)	52 (37.7)		
Preoperative chemotherapy, n (%)			χ² = 0.123	0.789
No	84 (69.4)	93 (67.4)		
Yes	37 (30.6)	45 (32.6)		
ASA physical status, n (%)			Z = −1.383	0.167
Grade II	78 (64.5)	100 (72.5)		
Grade III	43 (35.5)	38 (27.5)		

RTG, robotic gastrectomy group; LTG, laparoscopic gastrectomy group. IQR, interquartile range. No statistically significant between-group differences exist for any baseline characteristic shown.

### 3.2 Surgical outcomes and pathological characteristics

**Figure 6:**
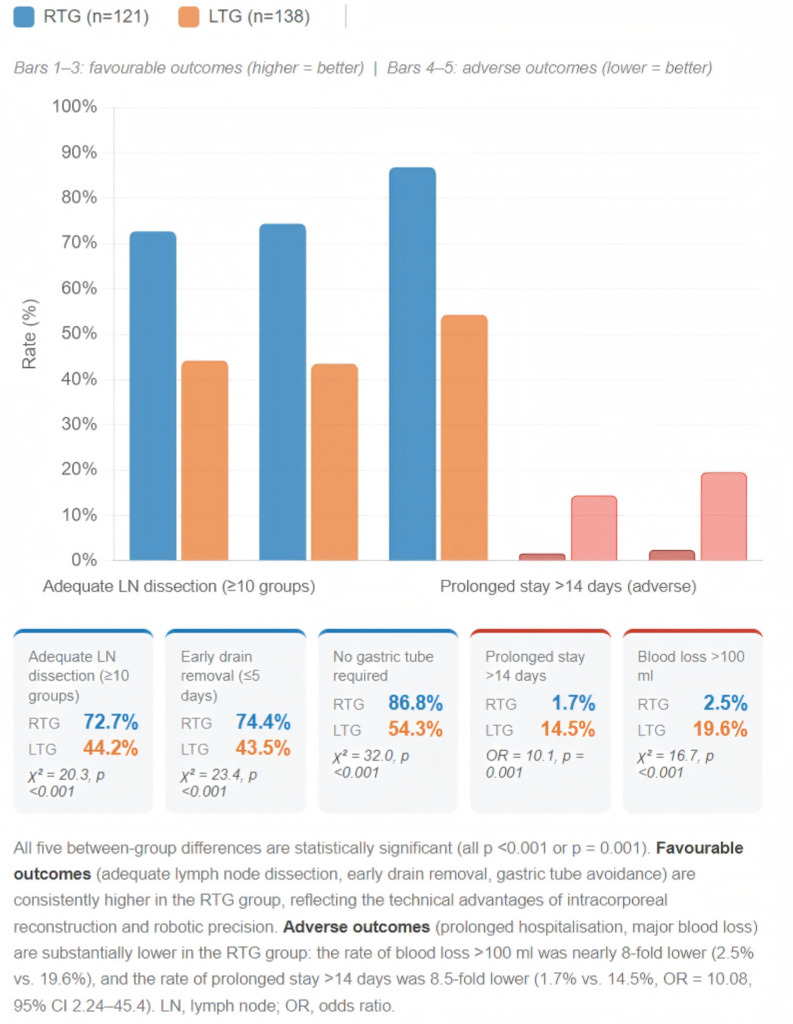
Clinically meaningful binary outcome rates: RTG versus LTG.

All patients completed surgery without intraoperative mortality. Two patients in the LTG required reoperation (1.4%) versus none in the RTG. Median operative time was comparable (RTG 260 vs. LTG 242.5 min, p=0.051); mean operative times were 264.0 ± 47.9 min (RTG) and 253.1 ± 71.9 min (LTG). Although median intraoperative blood loss was equal (50 ml each), the rate of clinically significant blood loss (>100 ml) differed markedly: only 2.5% of RTG patients (3/121) experienced >100 ml blood loss compared to 19.6% of LTG patients (27/138) (χ²=16.747, p<0.001), representing a nearly 8-fold difference in rate. Mean blood loss was 57.3 ± 50.4 ml (RTG) versus 94.4 ± 91.5 ml (LTG). The RTG group achieved a significantly higher median lymph node yield (10 vs. 9 groups, p<0.001). When evaluated against a clinically meaningful threshold, the proportion of patients achieving ≥10 lymph node groups — reflecting complete D2 dissection — was 72.7% (88/121) in the RTG group versus 44.2% (61/138) in the LTG group (χ²=20.318, p<0.001). A visual comparison of five clinically meaningful binary outcome rates across both groups is provided in **Figure 6**.

**Figure 7:**
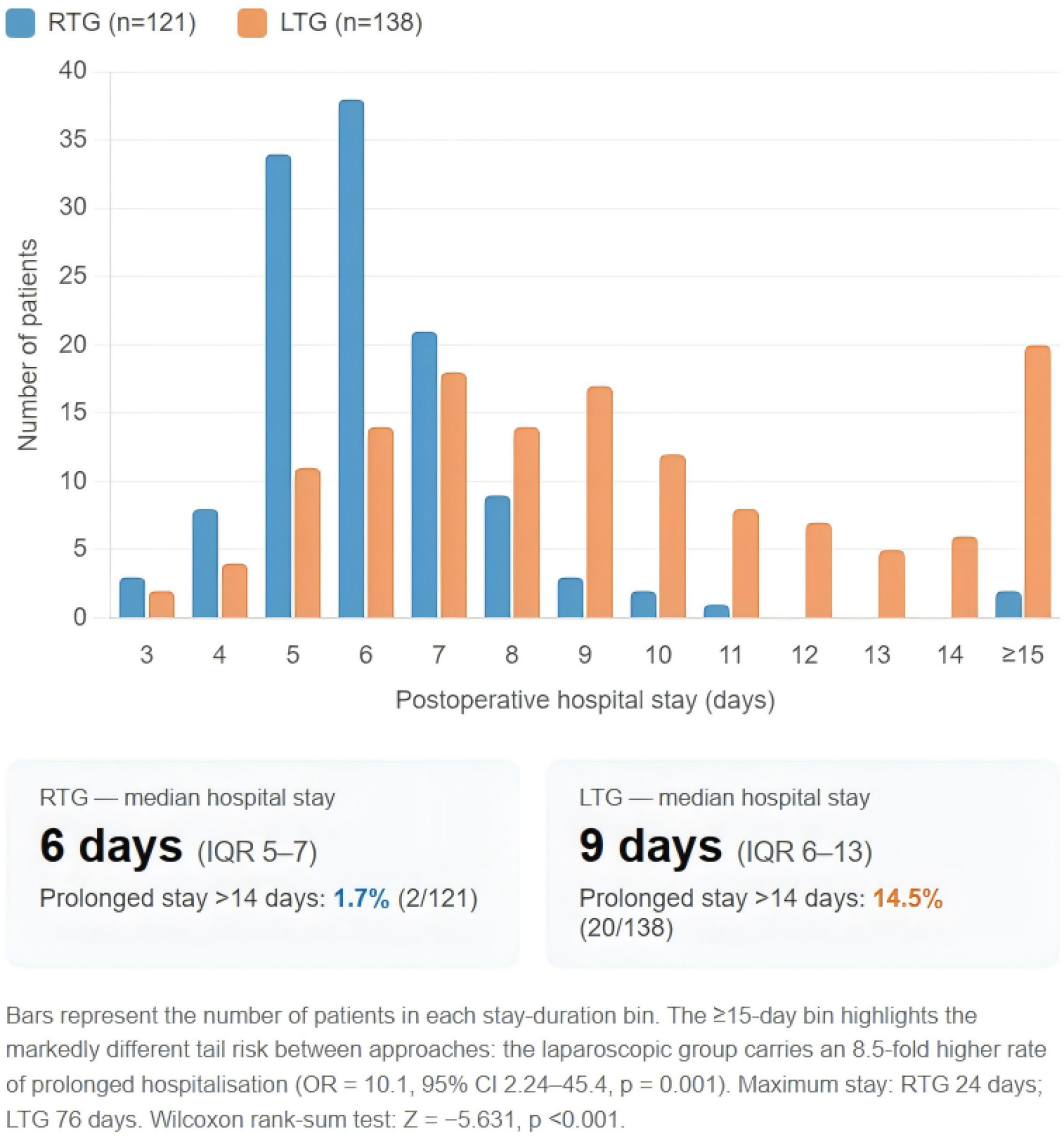
Distribution of postoperative hospital stay (days) in the RTG and LTG groups.

Pathological examination revealed a significant baseline imbalance in tumour stage (Stage I: 40.5% RTG vs. 21.7% LTG, p=0.003) and preoperative albumin (40.8 vs. 39.05 g/L, p<0.001). These differences are addressed through multivariate analysis (Section 3.5). The proportion of patients not requiring ICU transfer was significantly higher in the RTG group (95.0% vs. 82.6%, p=0.002). VAS pain scores were significantly lower in the RTG group (p<0.001). The rate of prolonged hospitalisation (>14 days) was 1.7% (2/121) in the RTG group versus 14.5% (20/138) in the LTG group (χ²=12.073, p=0.001; OR = 10.08, 95% CI 2.24–45.4); the maximum stay was 24 days (RTG) versus 76 days (LTG). The full distribution of postoperative hospital stay, illustrating the markedly different tail risk between the two groups, is shown in **Figure 7**. Detailed surgical outcomes and pathological characteristics are presented in **Table 2**.

**Table 2:** Surgical outcomes and pathological characteristics (RTG vs. LTG).

Variables	RTG (n=121)	LTG (n=138)	Statistic	P value
Surgical procedure, n (%)			χ² = 1.368	0.512
Distal gastrectomy	68 (56.2)	68 (49.3)		
Total gastrectomy	24 (19.8)	34 (24.6)		
Proximal gastrectomy	29 (24.0)	36 (26.1)		
Operative time, min (median, IQR)	260 (230, 295)	242.5 (195, 300)	Z = −1.951	0.051
Mean operative time (mean ± SD)	264.0 ± 47.9	253.1 ± 71.9	t = −1.46	0.146
Intraoperative blood loss, ml (median, IQR)	50 (50, 60)	50 (50, 100)	Z = −4.747	<0.001
Mean blood loss (mean ± SD)	57.3 ± 50.4	94.4 ± 91.5	t = −4.21	<0.001
Blood loss >100 ml, n (%)	3 (2.5)	27 (19.6)	χ² = 16.747	<0.001
Lymph node groups dissected (median, IQR)	10 (9, 10)	9 (6, 10)	Z = −5.730	<0.001
Adequate LN dissection (≥10 groups), n (%)	88 (72.7)	61 (44.2)	χ² = 20.318	<0.001
Reoperation, n (%)	0 (0)	2 (1.4)		0.500
ICU admission not required, n (%)	115 (95.0)	114 (82.6)	χ² = 9.730	0.002
VAS pain score, n (%)			Z = −5.322	<0.001
Grade 1–3 (mild)	103 (85.2)	75 (54.3)		
Grade 4–6 (moderate)	17 (14.0)	58 (42.1)		
Grade 7–10 (severe)	1 (0.8)	5 (3.5)		
Tumour size, cm (median, IQR)	2.8 (2.0, 4.0)	3.0 (2.2, 4.6)	Z = −2.513	0.012
Pathologic T stage, n (%)			Z = 9.349	0.040
T0–T1	47 (38.8)	32 (23.2)		
T2	12 (9.9)	12 (8.7)		
T3	50 (41.3)	74 (53.6)		
T4	12 (9.9)	20 (14.5)		
Pathologic N stage, n (%)			Z = −2.349	0.019
N0	59 (48.8)	53 (38.4)		
N1–N3	62 (51.2)	85 (61.6)		
Pathologic TNM stage, n (%)			Z = −3.022	0.003
Stage I	49 (40.5)	30 (21.7)		
Stage II	34 (28.1)	47 (34.1)		
Stage III	38 (31.4)	61 (44.2)		
Prolonged hospitalisation (>14 days), n (%)	2 (1.7)	20 (14.5)	χ² = 12.073	0.001

ICU, intensive care unit; VAS, visual analogue scale; LN, lymph node; IQR, interquartile range; SD, standard deviation.

### 3.3 Postoperative recovery and nutritional status

Postoperative recovery outcomes are summarised in **Table 3**. The RTG group demonstrated significantly faster recovery across all ERAS milestones: first liquid intake (6 vs. 42.75 h, p<0.001), first semi-liquid (50 vs. 82 h, p<0.001), first solid intake (97 vs. 139.5 h, p<0.001), first ambulation (20 vs. 38 h, p<0.001), first flatus (61.5 vs. 69.5 h, p=0.015), urinary catheter duration (14 vs. 23.5 h, p<0.001), and drain duration (5 vs. 6 days, p<0.001). Early drain removal (within 5 days) was achieved in 74.4% of RTG patients versus 43.5% of LTG patients (p<0.001). Median postoperative hospital stay was 6 days (RTG) versus 9 days (LTG) (p<0.001); mean stay was 6.48 ± 2.74 versus 9.98 ± 7.48 days. Indwelling gastric tube was required in only 13.2% of RTG patients versus 45.7% in the LTG group (p<0.001).

**Table 3:** Postoperative recovery indicators and laboratory values (RTG vs. LTG).

Variables	RTG (n=121)	LTG (n=138)	Statistic	P value
Time to first liquid intake, h (median, IQR)	6 (5, 10)	42.75 (6.00, 92.38)	Z = −7.227	<0.001
Time to first semi-liquid intake, h (median, IQR)	50 (43.25, 63.00)	82 (50.00, 149.25)	Z = −6.780	<0.001
Time to first solid intake, h (median, IQR)	97 (86.5, 118.0)	139.5 (96.75, 236.50)	Z = −6.487	<0.001
Time to first ambulation, h (median, IQR)	20 (16, 23)	38 (20.00, 59.25)	Z = −7.346	<0.001
Time to first flatus, h (median, IQR)	61.5 (42.0, 82.5)	69.5 (46.00, 104.25)	Z = −2.423	0.015
Urinary catheter duration, h (median, IQR)	14 (10.25, 19.50)	23.5 (16.0, 44.0)	Z = −6.623	<0.001
Abdominal drain duration, days (median, IQR)	5 (4, 6)	6 (4, 9)	Z = −4.747	<0.001
Drain removed within 5 days, n (%)	90 (74.4)	60 (43.5)	χ² = 23.4	<0.001
Postoperative hospital stay, days (median, IQR)	6 (5, 7)	9 (6, 13)	Z = −5.631	<0.001
Mean hospital stay (mean ± SD)	6.48 ± 2.74	9.98 ± 7.48	t = −4.87	<0.001
Prolonged stay >14 days, n (%)	2 (1.7)	20 (14.5)	χ² = 12.073	0.001
Indwelling gastric tube, n (%)	16 (13.2)	63 (45.7)	χ² = 31.984	<0.001
Preoperative laboratory values				
Albumin, g/L (median, IQR)	40.8 (38.2, 43.0)	39.05 (36.60, 41.93)	Z = −3.693	<0.001
Preop hypoalbuminaemia (<35 g/L), n (%)	9 (7.4)	17 (12.3)	χ² = 1.87	0.172
WBC, ×10^9^/L (median, IQR)	5.2 (4.16, 6.20)	5.15 (4.30, 6.27)	Z = −0.162	0.871
Haemoglobin, g/L (median, IQR)	127 (106, 142)	125 (103, 136)	Z = −1.516	0.130
CEA, ng/ml (median, IQR)	1.79 (1.15, 2.92)	2.17 (1.35, 3.12)	Z = −1.289	0.197
CA19-9, U/ml (median, IQR)	6.22 (2.78, 12.81)	4.78 (2.60, 13.83)	Z = −0.259	0.796
Postoperative day 1 laboratory values				
Albumin, g/L (mean ± SD)	39.32 ± 3.69	35.55 ± 4.83	t = −6.987	<0.001
Δ Albumin from baseline, g/L (median, IQR)	−2.0 (−4.4, 1.1)	−3.9 (−7.0, −0.4)	Z = −3.39	0.001
Electrolyte imbalance, n (%)	69 (58.0)	103 (76.9)	χ² = 10.325	0.002
Postoperative day 5 laboratory values				
Albumin, g/L (median, IQR)	39.85 (37.23, 43.70)	35.0 (32.0, 38.0)	Z = −7.408	<0.001
Δ Albumin from baseline, g/L (median, IQR)	−0.7 (−3.2, 1.8)	−3.2 (−6.7, −1.2)	Z = −5.12	<0.001
Electrolyte imbalance, n (%)	50 (52.6)	79 (66.9)	χ² = 4.517	0.036

Δ Albumin = postoperative value minus preoperative value (negative = decline from baseline). IQR, interquartile range; SD, standard deviation.

**Figure 8:**
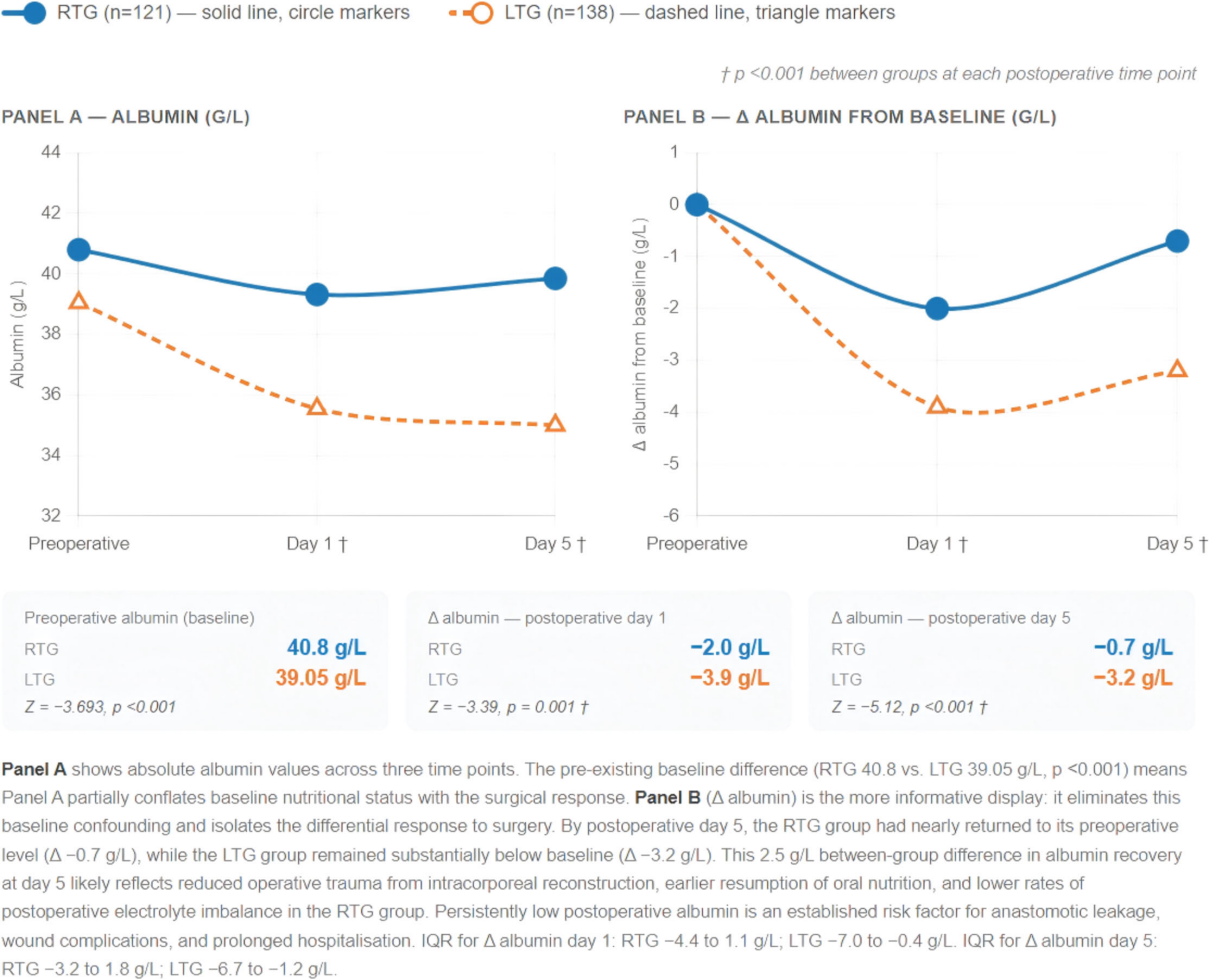
Postoperative serum albumin trajectory: absolute values (left) and change from preoperative baseline (right).

A novel analysis of the differential postoperative albumin trajectory revealed that the RTG group better preserved nutritional status throughout the recovery period. Δ albumin (postoperative minus preoperative) on day 1 was −2.0 g/L (IQR −4.4 to 1.1) in the RTG group versus −3.9 g/L (IQR −7.0 to −0.4) in the LTG group (p=0.001). By day 5, the RTG group had nearly returned to preoperative levels (Δ −0.7 g/L, IQR −3.2 to 1.8), while the LTG group remained substantially below baseline (Δ −3.2 g/L, IQR −6.7 to −1.2; p<0.001). This differential recovery of albumin likely reflects the reduced surgical trauma associated with intracorporeal reconstruction and the earlier resumption of oral nutrition in the RTG group. The albumin trajectory and the change-from-baseline (Δ albumin) plots are shown in **Figure 8**; the right panel (Δ albumin) is particularly informative as it removes the confounding effect of the pre-existing baseline difference between groups.

### 3.4 Postoperative complications

Complication data are shown in **Table 4**. The overall postoperative complication rate was 5.0% (RTG) versus 7.2% (LTG). The OR was 0.68 (95% CI 0.24–1.94, p=0.606), indicating no statistically significant difference. Post-hoc power analysis revealed that this comparison had only 11.6% power to detect the observed absolute difference of 2.2 percentage points at two-sided α=0.05. The wide 95% CI spans from a 76% reduction to a 94% increase in complication odds, and this result should be interpreted as statistically inconclusive rather than as evidence of equivalence. Clavien-Dindo classification did not differ significantly (p=0.257). No perioperative mortality occurred in either group.

**Table 4:** Postoperative complications and in-hospital mortality.

Variables	RTG (n=121)	LTG (n=138)	Statistic	P value
Overall complications, n (%)	6 (5.0)	10 (7.2)	χ² = 0.582	0.606
OR = 0.68 (95% CI 0.24–1.94); *post-hoc* power = 11.6%				
Clavien–Dindo grade, n (%)			Z = −1.134	0.257
Grade I–II	6 (100)	8 (80.0)		
Grade III–V	0 (0)	2 (20.0)		
Specific complications, n (%)				
Wound infection	1 (0.83)	2 (1.45)		1.000
Pulmonary infection	2 (1.65)	1 (0.72)		0.600
Intra-abdominal infection	1 (0.83)	4 (2.90)		0.376
Anastomotic leakage	1 (0.83)	1 (0.72)		1.000
Other complications	0 (0)	2 (1.45)		—
In-hospital mortality, n (%)	0 (0)	0 (0)	N/A	N/A

OR, odds ratio; CI, confidence interval. Post-hoc power calculated for two-sided comparison of proportions (α=0.05).

### 3.5 Multivariate analysis and subgroup analyses

Multivariate regression results are presented in **Table 5** for key recovery outcomes. After adjustment for pathological TNM stage, preoperative albumin, age, and BMI, the robotic approach remained independently and significantly associated with shorter postoperative hospital stay (β=−3.25 days, 95% CI −4.72 to −1.77, p<0.001), earlier first liquid intake (β=−41.71 h, 95% CI −53.04 to −30.37, p<0.001), earlier first ambulation (β=−16.45 h, 95% CI −20.97 to −11.92, p<0.001), and shorter drain duration (β=−1.79 days, 95% CI −2.52 to −1.06, p<0.001). In the logistic model, the robotic approach was not significantly associated with complication occurrence (OR = 0.88, 95% CI 0.30–2.64, p=0.825), consistent with the underpowered univariate comparison. These results confirm that the recovery advantages observed in univariate analysis cannot be attributed to the more favourable staging distribution of the RTG group.

**Table 5:** Multivariate regression: independent effect of robotic approach on postoperative outcomes (adjusted for TNM stage, preoperative albumin, age, and BMI).

Outcome (RTG vs. LTG, adjusted)	Coefficient	95% CI	P value
Linear regression outcomes (β = mean difference, RTG minus LTG)			
Post-op hospital stay (days)	β = −3.25	−4.72 to −1.77	<0.001
Time to first liquid intake (h)	β = −41.71	−53.04 to −30.37	<0.001
Time to first ambulation (h)	β = −16.45	−20.97 to −11.92	<0.001
Drain removal time (days)	β = −1.79	−2.52 to −1.06	<0.001
Logistic regression outcome (OR for complication occurrence)			
Overall complications	OR = 0.88	0.30 to 2.64	0.825

All models adjusted for: pathological TNM stage (categorical, Stage I as reference), preoperative serum albumin (continuous, g/L), age (continuous, years), BMI (continuous, kg/m²). β = unstandardised linear regression coefficient (negative = shorter/lower in RTG). OR = odds ratio from binary logistic regression.

**Figure 9:**
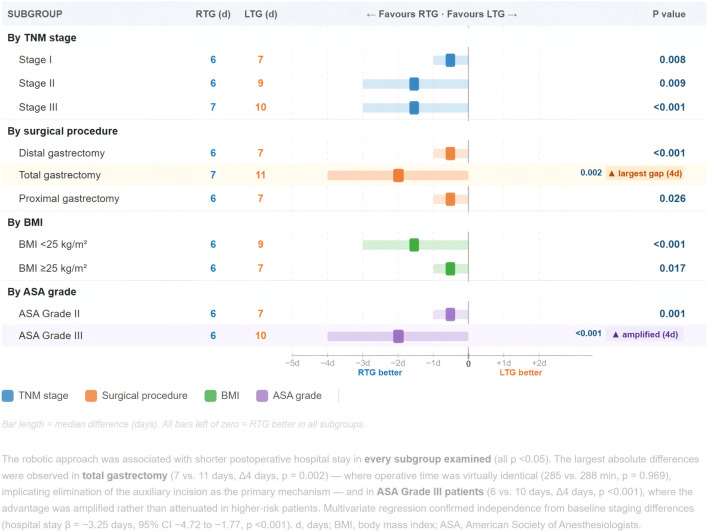
Subgroup consistency forest plot: postoperative hospital stay difference (RTG vs. LTG) across all patient subgroups.

**Table 6** and **Figure 9** present subgroup analysis of postoperative hospital stay across all patient subgroups. The RTG advantage in hospital stay was statistically significant in every stratum examined. The largest absolute difference was observed in total gastrectomy (7 vs. 11 days, p=0.002 — a 4-day gap), compared to 1-day differences in distal and proximal gastrectomy. Critically, operative time for total gastrectomy was virtually identical between groups (285 vs. 288 min, p=0.969), demonstrating that the recovery advantage in this subgroup is attributable specifically to the elimination of the auxiliary incision through intracorporeal reconstruction, not to a more expeditious operation. Among ASA Grade III patients (RTG n=43, LTG n=38), the advantage was amplified (6 vs. 10 days, p<0.001) rather than attenuated, with 0% versus 5.3% complication rates. The consistency of the RTG advantage across all 10 subgroup strata, as visually confirmed by **Figure 9**, provides compelling evidence that this benefit is not confined to any particular patient or disease subset.

**Table 6:** Subgroup analysis of postoperative hospital stay (days) stratified by TNM stage, surgical procedure, BMI, and ASA grade.

Subgroup	RTG n	RTG median (d)	LTG n	LTG median (d)	P value
By TNM stage					
Stage I	49	6	30	7	0.008
Stage II	34	6	47	9	0.009
Stage III	38	7	61	10	<0.001
By surgical procedure					
Distal gastrectomy	68	6	68	7	<0.001
Total gastrectomy	24	7	34	11	0.002
Proximal gastrectomy	29	6	36	7	0.026
By BMI					
BMI <25 kg/m²	85	6	104	9	<0.001
BMI ≥25 kg/m²	36	6	34	7	0.017
By ASA grade					
ASA Grade II	78	6	100	7	0.001
ASA Grade III	43	6	38	10	<0.001

All comparisons by Wilcoxon rank-sum test (two-sided). Entire table is new analysis added in this revision.

## 4 Discussion

This study compared robotic versus laparoscopic radical gastrectomy within a uniform ERAS protocol, employing both univariate comparisons and multivariate analyses adjusted for key confounders. The principal findings are: (1) robotic gastrectomy is independently associated with faster postoperative recovery across all ERAS milestones after multivariate adjustment; (2) robotic surgery substantially reduces the rate of prolonged hospitalisation (1.7% vs. 14.5%); (3) clinically significant blood loss (>100 ml) is markedly less frequent with robotic surgery (2.5% vs. 19.6%); (4) robotic surgery achieves higher rates of adequate lymph node dissection (≥10 groups: 72.7% vs. 44.2%); (5) the recovery advantage is largest in total gastrectomy, where operative time is equal; and (6) robotic surgery better preserves postoperative nutritional status as measured by serum albumin trajectory.

### 4.1 Methodological rigour and comparison with prior RCTs

The most important methodological advance of the present study is the application of multivariate regression to control for the significant baseline imbalance in pathological TNM stage. After adjustment for TNM stage, preoperative albumin, age, and BMI, the robotic approach retained independent and statistically significant associations with all four primary continuous outcomes (all p<0.001). These adjusted results are directionally consistent with the randomised trial by Lu et al., which reported shorter postoperative hospital stay and earlier recovery with robotic distal gastrectomy (15), and with the 3-year follow-up from the same group confirming non-inferior oncological outcomes (16). Ojima et al.'s Japanese RCT similarly showed lower pancreatic injury markers and comparable short-term complication rates with robotic surgery (14). The magnitude of the adjusted hospital-stay difference in our cohort (β = −3.25 days) is numerically larger than that reported in these pure distal-gastrectomy RCTs, likely reflecting the greater representation of total gastrectomy cases in our series, where intracorporeal reconstruction confers its largest advantage.

Our findings are also supported at a population level by the Guerrini et al. meta-analysis of over 17,000 patients (18), a recent Bayesian network meta-analysis placing robotic gastrectomy as the optimal approach for short-term recovery (19), and the Li et al. Chinese multicentre cohort of 5,402 patients—currently the largest real-world comparison available—which reported statistically significant advantages for the robotic approach in blood loss, recovery milestones, and perioperative morbidity (20). European observational data from van Boxel et al. further confirm that these findings generalise beyond Asian high-volume centres (22).

### 4.2 Prolonged hospitalisation as an economically relevant endpoint

The difference in prolonged-hospitalisation rates (>14 days) deserves particular attention: 14.5% of LTG patients experienced stays exceeding two weeks, compared with only 1.7% of RTG patients (OR = 10.1, p=0.001). Prolonged hospitalisation represents a qualitatively different clinical outcome from a difference in median stay—it captures patients who encountered a substantially complicated postoperative course, with implications for patient welfare, bed occupancy, nursing burden, and hospital costs. From a health-system perspective, the 12.8 percentage-point absolute reduction in prolonged-stay rate may be the most economically relevant finding in this study. The maximum stay of 76 days in the LTG group versus 24 days in the RTG group underscores the difference in tail risk between the two approaches—a signal largely invisible in conventional median-based comparisons but directly relevant to real-world resource allocation, as noted in recent European multicentre analyses of locally advanced gastric cancer (23).

### 4.3 Clinically significant blood loss and its oncological implications

The categorical analysis of blood loss provides a more clinically interpretable account of the haemostatic advantage of robotic surgery than the raw Wilcoxon comparison. The near-identical medians (50 ml each) yet highly significant difference in the >100 ml rate (2.5% vs. 19.6%, p<0.001) reveals the true pattern: robotic surgery essentially eliminates major haemorrhagic events, while laparoscopic surgery carries a persistent risk of larger bleeding episodes—most likely during technically demanding peripancreatic and splenic hilar dissection, consistent with the intraoperative adverse event patterns identified in a pooled analysis of two Chinese laparoscopic RCTs (24).

Minimising intraoperative blood loss is not merely a technical goal but an oncologically meaningful one. A 2022 meta-analysis of 4,653 gastric cancer patients demonstrated that larger intraoperative blood loss was independently associated with a higher complication rate (OR = 1.94, 95% CI 1.44–2.61) and longer operative time, as well as adverse long-term survival signals (25). Allogeneic blood transfusion—which becomes substantially more likely when intraoperative losses exceed 100 ml—has been linked to anti-tumour immunosuppression, peritoneal tumour spillage, and worse oncological outcomes in gastric cancer (26). The near-elimination of major bleeding events with robotic surgery may therefore have downstream implications beyond perioperative recovery.

### 4.4 The mechanistic signal from total gastrectomy

The subgroup analysis by surgical procedure reveals a mechanistically informative pattern. In total gastrectomy—where Roux-en-Y esophagojejunostomy requires the most complex reconstruction and the largest auxiliary incision in laparoscopic surgery—the RTG group had a 4-day shorter hospital stay (7 vs. 11 days, p=0.002) despite identical operative time (285 vs. 288 min, p=0.969). This combination of equal operative time and dramatically different recovery provides the strongest evidence that the recovery benefit is specifically attributable to the elimination of the auxiliary incision, not to general operative efficiency. The same pattern has been reported by Hikage et al. in a Japanese propensity-matched total gastrectomy cohort, where the robotic advantage was most pronounced in the total gastrectomy subgroup (27), and by Uyama et al.'s multi-institutional prospective study, which similarly identified total gastrectomy as the procedure in which robotic surgery delivers the greatest clinical value (28). Robotic surgery should therefore be particularly prioritised for patients requiring total gastrectomy.

### 4.5 Postoperative albumin trajectory as a validated recovery biomarker

The differential postoperative albumin trajectory offers mechanistic insight into how robotic surgery reduces physiological stress. As shown in **Figure 8**, the smaller decline in serum albumin in the RTG group (Δ −2.0 vs. Δ −3.9 g/L on day 1; Δ −0.7 vs. Δ −3.2 g/L on day 5) quantifies a reduced surgical stress response. The postoperative decrease in albumin (ΔAlbumin) has been systematically validated as an early predictor of postoperative complications across 16 studies in gastrointestinal surgery, with thresholds in the range of 5–11 g/L identifying patients at substantially higher risk (29). By this metric, the RTG group's mean day-1 ΔAlbumin of −2.0 g/L remains well below the typical risk threshold, while the LTG group's Δ −3.9 g/L approaches it—providing an objective, laboratory-anchored explanation for the differential complication and prolonged-stay rates observed clinically.

The RTG group's near-complete recovery of albumin to baseline by day 5 (Δ −0.7 g/L) likely reflects a combination of (1) lower operative trauma from the smaller surgical footprint, (2) earlier resumption of oral protein intake—postoperative gastrointestinal dysfunction being a well-documented driver of delayed recovery after major abdominal surgery (30)—and (3) lower rates of electrolyte imbalance. Lower postoperative albumin is an established risk factor for anastomotic leakage, wound-healing complications, and prolonged hospitalisation; the robotic group's preserved nutritional status therefore likely contributes directly to the earlier discharge observed in this cohort.

### 4.6 Drain duration as the new rate-limiting step under ERAS

**Figure 10:**
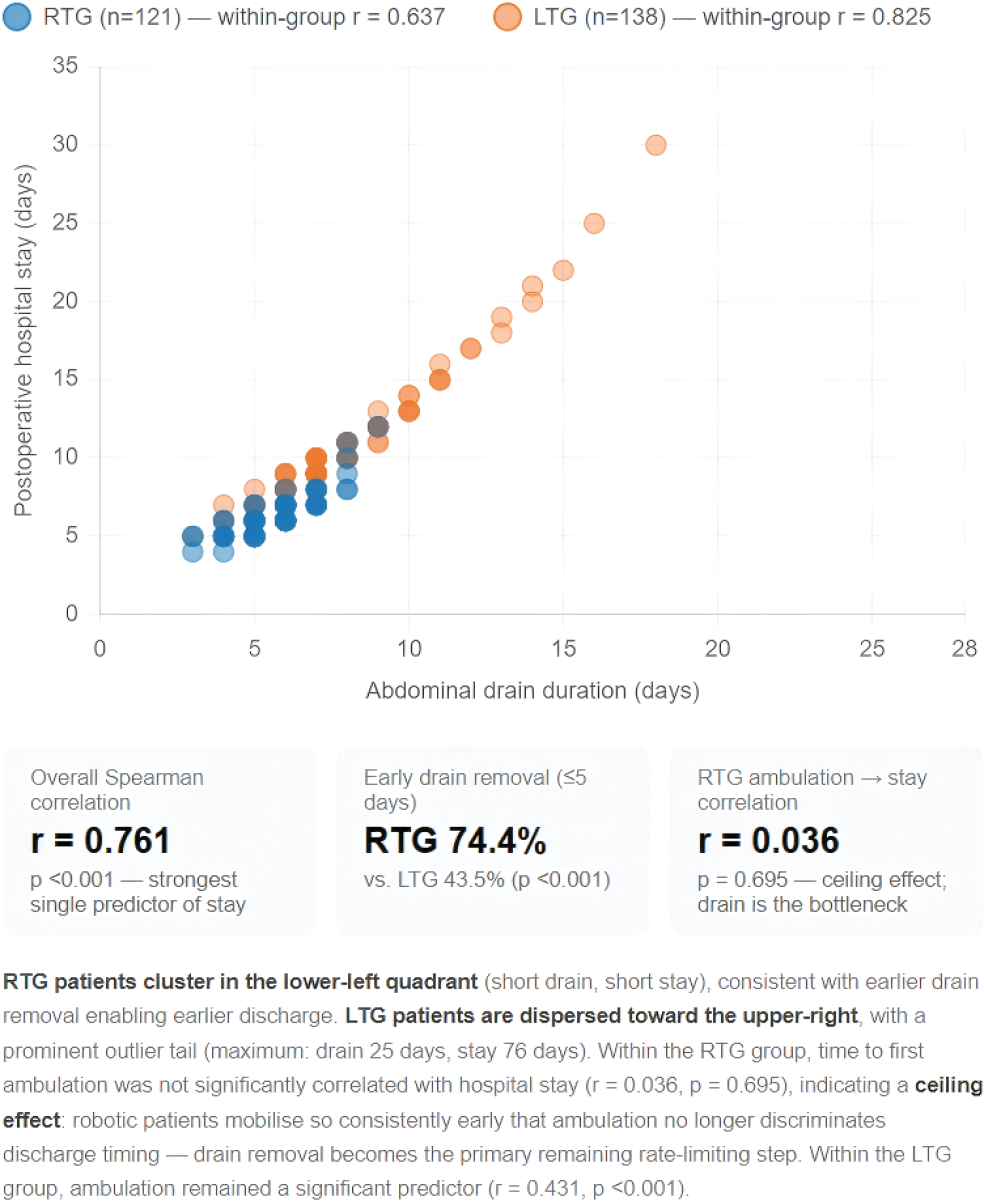
Scatter plot: abdominal drain duration versus postoperative hospital stay (overall Spearman r = 0.761, p<0.001).

Correlation analysis identified abdominal drain duration as the strongest predictor of hospital stay in this cohort (Spearman r = 0.761, p<0.001)—substantially stronger than first oral intake (r = 0.586) or first ambulation (r = 0.369). As illustrated in **Figure 10**, RTG patients cluster tightly in the lower-left quadrant of the drain-versus-stay scatter plot, while LTG patients disperse toward the upper-right with a prominent tail of prolonged-drain/prolonged-stay outliers. The RTG group achieved drain removal within 5 days in 74.4% of patients versus 43.5% in the LTG group. Intriguingly, within the RTG group first ambulation time was not significantly correlated with hospital stay (r = 0.036, p = 0.695), whereas within the LTG group the same correlation was 0.431 (p < 0.001). This pattern suggests a "ceiling effect" in the RTG group: robotic patients ambulate so consistently and early that mobilisation no longer discriminates who is discharged sooner. Drain removal has thus emerged as the new rate-limiting step in ERAS pathways for robotic gastrectomy—an observation that aligns with the ERAS Society gastrectomy guideline's emphasis on minimising unnecessary drainage (6) and identifies an appropriate target for further optimisation at high-volume robotic centres.

### 4.7 The inconclusive complication comparison

The overall complication rate did not differ significantly (5.0% vs. 7.2%, OR = 0.68, 95% CI 0.24–1.94, p = 0.606). Post-hoc power analysis revealed only 11.6% power to detect the observed difference, and the 95% CI spans both a 76% reduction and a 94% increase in complication odds. This result is statistically inconclusive and should not be interpreted as equivalence. The numerical trend of fewer high-grade (Clavien-Dindo ≥III) complications in the RTG group (0 vs. 2) is consistent with published RCTs (14, 15, 17) and the Guerrini meta-analysis (18), but cannot be confirmed with the present sample size. Definitive assessment of complication endpoints will require adequately powered multicentre randomised trials.

### 4.8 Limitations

Several limitations should be acknowledged. First, as a single-centre retrospective analysis, selection bias and unmeasured confounding cannot be fully excluded. The significant baseline imbalance in TNM stage reflects real-world selective adoption of robotic surgery and was addressed by multivariate adjustment and subgroup analyses, but residual confounding remains possible. Second, individual patient-level ERAS compliance was not prospectively audited; although all patients were managed under the same institutional protocol implemented since 2016, we cannot rule out inter-patient variation in compliance. Third, the complication comparison was substantially underpowered. Fourth, long-term oncological outcomes were not assessed, and the relationship between superior short-term recovery and long-term benefit remains to be established prospectively. The recent 3-year disease-free survival data from Lu et al. (16) provides encouraging early evidence that the short-term recovery advantages of robotic gastrectomy do not come at the cost of oncological safety, but longer follow-up in larger cohorts is needed.

## 5 Conclusion

Robotic radical gastrectomy within a uniform ERAS protocol is independently associated with superior short-term postoperative recovery — including shorter hospitalisation, earlier oral intake, earlier mobilisation, faster return of nutritional status, and a markedly lower rate of prolonged hospitalisation — compared to laparoscopic surgery, with this advantage confirmed by multivariate adjustment and consistent across all TNM stages, procedure types, BMI categories, and ASA grades. The robotic approach also achieves higher rates of clinically adequate lymph node dissection and virtually eliminates major intraoperative blood loss events. The largest absolute benefit is observed in total gastrectomy, where equal operative time combined with a 4-day shorter stay demonstrates that the advantage stems specifically from intracorporeal reconstruction. Multi-centre prospective randomised trials incorporating long-term oncological endpoints, ERAS compliance monitoring, and health-economic analyses are required to provide definitive evidence.

## Data Availability

The original contributions presented in the study are included in the article/supplementary material. Further inquiries can be directed to the corresponding authors.
